# Chemical Composition and Biological Activities of the Essential Oil of *Skimmia laureola* Leaves

**DOI:** 10.3390/molecules20034735

**Published:** 2015-03-16

**Authors:** Barkatullah, Muhammad Ibrar, Naveed Muhammad, Vincenzo De Feo

**Affiliations:** 1 Department of Botany, University of Peshawar, Peshawar 25000, Pakistan; E-Mails: bu_barq@yahoo.com (B.); ibrarm200@yahoo.com (M.I.); 2 Department of Pharmacy, Abdul Wali Khan University, Mardan 23200, Pakistan; E-Mail: drnaveedrph@gmail.com; 3 Department of Pharmacy, University of Salerno, Fisciano 84084, Italy

**Keywords:** *Skimmia laureola*, antispasmodic activity, antibacterial activity, antifungal activity, phytotoxic activity, cytotoxic activity

## Abstract

The composition of the essential oil from leaves of *Skimmia laureola* was determined by GC and GC-MS. Twenty-eight components were identified, accounting for 93.9% of the total oil. The oil is mainly composed of monoterpenes (93.5%), of which monoterpene hydrocarbons and oxygenated monoterpenes represent 11.0% and 82.5%, respectively. Sesquiterpenes constitute only 0.3% of the total oil. Linalyl acetate is the main component (50.5%), with linalool (13.1%), geranyl acetate (8.5%) and *cis*-*p*-menth-2-en-1-ol (6.2%) as other principal constituents. The essential oil showed a significant antispasmodic activity, in a dose range of 0.03–10 mg/mL. The essential oil also possesses antibacterial and antifungal activities against some pathogenic strains. The phytotoxic and cytotoxic activities were also assessed.

## 1. Introduction

*Skimmia laureola* (DC.) Decne (Rutaceae) is an aromatic shrub distributed from northern China to the Northern Himalayas; its leaves give off an aromatic smell when crushed. Traditionally, the plant is used in folk and cultural practices: the smoke of dried leaves is considered useful to ward off evils [1] and leaves are arranged in garlands and considered sacred [2]. In traditional medicine the leaves are employed as an antitussive and, when dried and crushed, as a veterinary anthelmintic, and as an insecticide and pesticide. The smoke of the dried leaves is used to clear the nasal tract and for cold, fever and headache treatment [3]. Previous phytochemical studies reported the composition of the essential oil from *Skimmia laureola* from different areas [4,5,6,7,8]. Triterpenoids [9,10], alkaloids and coumarins have also been reported [11] in the plant. The essential oil has been evaluated for its antimicrobial and insect-repellent activities [4,7,12], and for acute toxicity, antinociceptive, antipyretic and anticonvulsant properties [13]. Quinoline alkaloids isolated from *S. laureola* have shown cholinesterase inhibiting and calcium blocking properties [10] and tyrosinase inhibiting activity [14]. Extracts of the plant have demonstrated antipyretic and antinociceptive [15], antifungal [16] and mutagenic activities [17]. There is limited research on its essential oil as a flavor or fragrance, so in this paper we report the composition of the essential oil from the leaves of *Skimmia laureola* collected in Pakistan and its possible antispasmodic, antibacterial, antifungal, phytotoxic and cytotoxic effects.

## 2. Results and Discussion

Table 1 reports the composition of essential oil from leaves of *Skimmia laureola*. On a fresh weight basis, the yield of the essential oil was 0.7%. Twenty-eight components were identified, accounting for 93.9% of the total oil. The oil is mainly composed by monoterpenes (93.5%), of which oxygenated monoterpenes represent 82.5% of the total oil. Sesquiterpenes constitute only 0.3% of the total oil. Linalyl acetate is the main component (50.5%), with linalool (13.1%), geranyl acetate (8.5%) and *cis*-*p*-menth-2-en-1-ol (6.2%) as principal constituents. This composition agrees with previously published data [5,6,7,8] and seems to also be characteristic of other *Skimmia* essential oils, such as S*kimmia anquetilia* N. P. Taylor & Airy Shaw [18].

**Table 1:** Composition of the essential oil from leaves of *Skimmia laureola.*

	Ki ^a^	Ki ^b^	%	Identification ^c^
(−)-Camphene	953	1076	T ^d^	1, 2, 3
Δ3-Carene	1008	1173	T	1, 2, 3
Caryophyllene oxide	1581	2008	T	1, 2, 3
Geranyl acetate	1379	1765	8.5	1, 2
*cis*-*p*-Menth-2-en-1-ol	1128	1638	6.2	1, 2
Linalyl acetate	1253	1665	50.5	1, 2
β-Myrcene	986	1162	3.4	1, 2, 3
(*Z*)-β-Ocimene	1034	1243	2.3	1, 2
α-Pinene	921	1032	2.2	1, 2, 3
Linalool	1096	1553	13.1	1, 2, 3
Neryl acetate	1367	2097	1.7	1, 2
(*E*)-β-Ocimene	1044	1262	1.6	1, 2
Sabinene	966	1132	1.0	1, 2
*p*-Menth-1-en-8-ol, acetate	1346	1738	1.0	1, 2
Geraniol	1255	1857	0.9	1, 2
β-Phellandrene	1029	1218	0.3	1, 2, 3
β-Pinene	978	1118	0.1	1, 2, 3
1,8-Cineole	1024	1213	0.1	1, 2, 3
Terpinolene	1083	1265	0.1	1, 2
Terpinen-4-ol	1173	1611	0.1	1, 2, 3
Octyl acetate	1208	1436	0.1	1, 2
Citral	1270	1727	0.1	1, 2
Bornyl acetate	1284	1591	0.1	1, 2
*cis*-Limonene oxide	1132	1450	0.1	1, 2
Caryophyllene	1408	1666	0.1	1, 2
α-Limonene diepoxide	1345		0.1	1, 2
γ-Elemene	1436	1650	0.1	1, 2
*trans*-Nerolidol	1565	2050	0.1	1, 2
**Total**			**93.9**	
**Monoterpene Hydrocarbons **			**11.0**	
**Oxygenated Monoterpenes**			**82.5**	
**Sesquiterpene Hydrocarbons **			**0.2**	
**Oxygenated Sesquiterpenes**			**0.1**	
**Other compounds**			**0.1**	

Notes: ^a^: Kovats retention index determined relative to the t_R_ of a series of *n*-alkanes (C_10_–C_35_) on HP-5 MS column. ^b^: Kovats retention index determined relative to the t_R_ of a series of *n*-alkanes (C_10_–C_35_) on HP Innowax. ^c^: 1 = Kovats retention index, 2 = mass spectrum, 3 = co-injection with authentic compound. ^d^: T = trace ( <0.1%).

Table 2 reports the antibacterial activity of three different doses of *S. laureola* essential oil. It was found active against five of the tested bacterial strains. *M. luteus* and *S. viridans* were found to be more susceptible, whereas no activity was found against *P. aeruginosa* and *B. subtilis*.

**Table 2:** Antibacterial effects of the essential oil of the leaves of *Skimmia laureola* (SVO).

Microorganism	SVO 125 µg/mL	SVO 250 µg/mL	SVO 500 µg/mL	DMSO	Imipenem
*M. luteus*	20.0 ± 1.33	21.2 ± 1.21	23.5 ± 1.87	---	28 ± 0.23
*E. coli*	11.0 ± 1.58	10.2 ± 0.87	11.3 ± 1.67	---	30 ± 0.10
*S. aureus*	13.0 ± 1.88	13.8 ± 0.95	14.0 ± 1.40	---	24 ± 0.65
*P. multocida*	15.8 ± 1.41	15.6 ± 1.98	16.3 ± 1.76	---	32 ± 0.24
*P. aeruginosa*	---	---	---	---	---
*B. subtilis*	---	---	---	---	---
*S. viridans*	15.3 ± 0.92	16.2 ± 2.23	18.5 ± 1.45	---	22 ± 0.45

Data are the mean ± SEM of three replicates.

The presence of high percentages of linalyl acetate and linalool in the oil can explain this activity. In fact, these compounds have been reported for their antibacterial properties, due to their ability to cross cell membranes, penetrating into the interior of the cell and interacting with critical intracellular sites [19]. 

The essential oil was evaluated for its antifungal activity (Table 3). All the tested fungal strains were inhibited in a concentration-dependent manner. The maximum inhibitory effect was observed for *C. albicans*, *A. flavus* and *T. longifusis*, at a dose of 125 µg/mL. Essential oils have been reported as antifungal agents and monoterpene-rich essential oils were active, particularly against *Candida* species [20]. Moreover, linalool is reported as an antifungal agent against several strains [21] and linalyl acetate has been reported for its activity against several *Candida* species [22].

**Table 3:** Antifungal effect of the essential oil of the leaves of *Skimmia laureola* (SVO).

	Percent Inhibition of Mycelia Growth				
Microorganism	SVO 125 µg/mL	SVO 250 µg/mL	SVO 500 µg/mL	DMSO	Miconazole
*T. longifusis*	62.66 ± 1.34	63.45 ± 1.45	67.65 ± 1.40	-	70.98 ± 0.40
*C. albicans*	67.32 ± 0.90	77.0 ± 1.56	83.87 ± 1.98	-	89.87 ± 0.22
*F. solani*	45.64 ± 1.40	56.7 ± 1.21	62.96 ± 1.11	-	73.98 ± 1.87
*M. canis*	30.37 ± 1.97	32.33 ± 1.17	50.00 ± 1.60	-	94.87 ± 0.99
*A. flavus*	64.45 ± 1.98	67.77 ± 1.88	70.97 ± 1.66	-	20.56 ± 0.65
*C. glabrata*	28.00 ± 1.14	61.33 ± 1.40	82.35 ± 1.49	-	93.98 ± 0.76

Data are the mean ± SEM of three replicates.

The essential oil was found to be phytotoxic in the *Lemna minor* test, with an LD_50_ of 46.09 μg/mL (Table 4). A significant dose-dependent cytoxicity was observed for all the doses tested (Table 5). The essential oil showed outstanding cytotoxic activity with an LD_50_ value of 11.01 µg/mL. Significant brine shrimp mortality was found at 10 µg/mL. Essential oils and monoterpenoids are well known as phytotoxic agents [23,24]. Linalool and linalyl acetate showed moderate inhibitory effects against *Raphanus sativus* L. and *Lepidium sativum* L. germination, but considerable inhibition against radical elongation of these test species [25]. 

**Table 4:** Phytotoxic effects of the essential oil of the leaves of *Skimmia laureola*.

Essential Oil	Number of Fronds	Living Fronds	Dead Fronds
10 µg/mL	52	30 ± 0.22	22 ± 1.89
100 µg/mL	52	21 ± 1.11	31± 1.99
1000 µg/mL	52	22 ± 1.00	30 ± 1.40

Data are the mean ±SEM of three replicates.

**Table 5:** Cytotoxic effects of the essential oil of the leaves of *Skimmia laureola*.

Essential Oil	Number of Brine Shrimps	Living Brine Shrimps	Dead Brine Shrimps
10 µg/mL	30	8 ± 1.34	22 ± 1.98
100 µg/mL	30	3 ± 1.00	27 ± 1.56
1000 µg/mL	30	0.9 ± 1.98	29.1 ± 1.75

Data are the mean ± SEM of three replicates.

Some essential oil have been reported for their toxicity in brine shrimp test [26]. It is of importance that data obtained by this test in most cases correlates reasonably well with cytotoxicity and anti-tumor properties [27].

**Figure 1:**
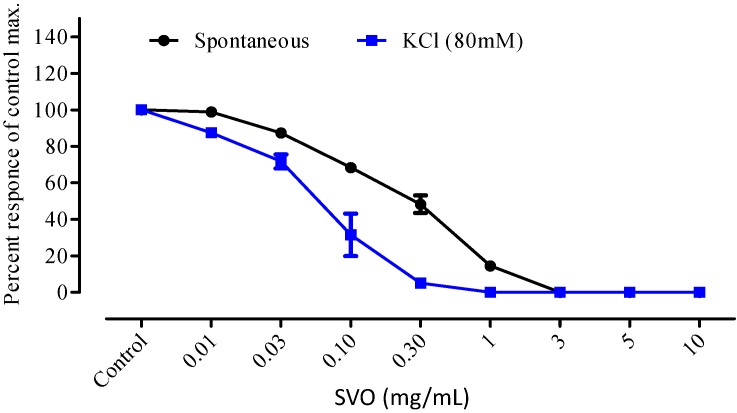
Dose response curve of *Skimmia laureola* essential oil (SVO) on isolated rabbit’s jejunum preparations. All values are the mean ± SEM, n = 5.

**Figure 2:**
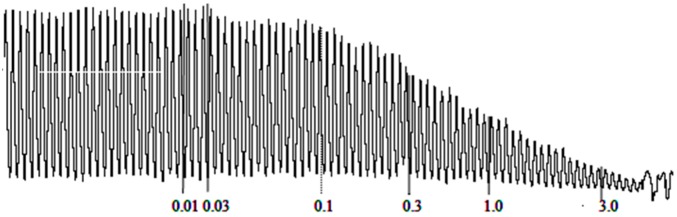
Effect of *Skimmia laureola* essential oil (mg/mL) on isolated rabbit jejuum.

It is evident from Figure 1 and Figure 2 that the essential oil of *S. laureola* significantly relaxed the contracted smooth muscles, in a dose-dependent manner. The spasmolytic effect of the oil started at 0.03 mg/mL and was 100% at a dose of 10 mg/mL.

The essential oil showed a remarkable antispasmodic activity, in spontaneous and KCl-induced contraction in isolated rabbit jejunum. The contraction of smooth muscle of rabbit jejunum is due to an increased concentration of free calcium in the cytoplasm, which stimulates the chemical mediators responsible for the contraction. This increase in calcium level may be either due to influx via voltage-dependent calcium channels or a direct release of calcium from the endoplasmic reticulum. Thus, a periodic depolarization is created due to high speed action potential. When there is an increase of potassium concentration, the contraction of the smooth muscle will increase due to rapid action potential. When the calcium channel is blocked through a calcium channel blocker agent, the contracted smooth muscle will relax. The muscle relaxation caused by *S. laureola* essential oil suggests that its possible mode of action is either by blocking the release of stored calcium from the sarcoplamic reticulum or by blocking the calcium channels.

## 3. Experimental Section 

### 3.1. Plant Collection

*Skimmia laureola* was collected in Patrak (Upper Dir), Pakistan, and shortly quickly at 4 °C in the dark. The plant was identified by Dr. Barkatullah and a voucher specimen (Bot. 8815) is kept in the herbarium of the Department of Botany, University of Peshawar, Pakistan. 

### 3.2. Isolation of Volatile Oil

One hundred g of dried leaves of *S. laureola* were ground in a Waring blender and then subjected to hydrodistillation for 3 h, according to the standard procedure described in the *European Pharmacopoeia* [28]. The oil was solubilized in *n*-hexane, filtered over anhydrous sodium sulfate and stored under N_2_ at +4 °C in the dark until tested and analyzed.

### 3.3. GC-FID and GC/MS Analyses and Identification of Essential Oil Components

Analytical gas chromatography was carried out on a Perkin-Elmer Sigma-115 gas chromatograph equipped with a FID and a data handling processor. The separation was achieved using a HP-5MS fused-silica capillary column (30 m × 0.25 mm i.d., 0.25 μm film thickness). Column temperature: 40 °C, with 5 min initial hold, and then to 270 °C at 2 °C/min, 270 °C (20 min); injection mode splitless (1 μL of a 1:1000 *n*-hexane solution). Injector and detector temperatures were 250 and 290 °C, respectively. Analysis was also run by using a fused silica HP Innowax polyethylene glycol capillary column (50 m × 0.20 mm i.d., 0.25 μm film thickness). In both cases, helium was used as carrier gas (1.0 mL/min). GC/MS analysis was performed on an Agilent 6850 Ser. II apparatus, fitted with a fused silica DB-5 capillary column (30 m × 0.25 mm i.d., 0.33 μm film thickness), coupled to an Agilent Mass Selective Detector MSD 5973; ionization energy voltage 70 eV; electron multiplier voltage energy 2000 V. Mass spectra were scanned in the 40–500 amu range, scan time was 5 scans/s. Gas chromatographic conditions were as reported above; transfer line temperature, 295 °C. Most constituents were identified by gas chromatography by comparison of their Kovats retention indices (Ri) [determined relative to the *t*R of *n-*alkanes (C_10_–C_35_)], with either those of the literature [29] or by comparison of mass spectra on both columns with those of authentic compounds available in our laboratories by means of the NIST 02 and Wiley 275 libraries [30,31,32,33]. The component relative concentrations were obtained by peak area normalization. No response factors were calculated.

### 3.4. Antibacterial Activity

Antibacterial activity was measured by the agar well diffusion method, against four Gram (+) [*Micrococcus luteus* ATCC 9341, *Staphylococcous aureus* ATCC 25923, *Bacillus subtilis* ATCC 6633, *Streptococcus viridians* ATCC 10556] and three Gram (−) [*Escherichia coli* ATCC25922, *Pasteurella multocida* (clinical isolate), *Psuedomonas aeruginosa* ATCC 27853] strains, purchased from Schazo Laboratories (Lahore, Pakistan) and Schering Pharmaceutical Industries Ltd.Lahore, Pakistan). Bacterial strains were first cultured on nutrient broth and incubated for 24 h. Nutrient agar was melted, cooled to 40 °C and poured into sterilized Petri dishes. Four-height hold bacterial culture was spread on the surface of nutrient agar. The experiment was repeated thrice turning the plate 60° between each streaking. About l00 µL of 3 mg/mL of essential oil dissolved in DMSO was then added to the wells. Other wells were supplemented with DMSO and 10 µg imipenem, used as positive and negative control, respectively. The plates were then incubated for 24 h at 37 °C. The plates were then observed for zones of inhibition, measuring the inhibition zones by using a caliper, mm/inch reading scale, precision 0.05 mm. All the experiments were conducted in triplicate.

### 3.5. Anti-Fungal Activity

The antifungal activity was assayed against six fungal strains: *Tricophyton longifusis* (clinical isolate), *Candida albicans* (ATCC 2091), *Fusarium solani* (ATCC 11712), *Microsporum canis* (ATCC 111622), *Aspergillus flavus* (ATCC 32611), and *Candida glabrata* (ATCC 90030). Twenty four mg of essential oil was dissolved in 1 ml sterile DMSO serving as a stock solution. Four mL of Sabouraud dextrose agar (SDA) growth media was transferred to each screw capped tube, under sterile conditions and autoclaved at 121 °C for 15 min. These tubes were then allowed to cool to 50 °C and 400 µg/mL test sample were added to the SDA tubes, which were then allowed to solidify at room temperature. DMSO and miconazole were used as negative and positive controls, respectively. The tubes were incubated at 28 ± 1 °C for 7 days. Cultures were observed twice weekly during incubation. Growth in the media was estimated by measuring the linear growth (mm) by .using a caliper, mm/inch reading scale, precision 0.05 mm. The percentage inhibition of fungal growth was calculated as follows:

% Mycelia inhibition = Gn − Gt/Gn × 100

where Gn = normal mycelial growth, Gt = mycelial growth in the test.

### 3.6. Phytotoxicity

The phytotoxic activity of the essential oil was evaluated using *Lemna minor* as test species, as previously reported [34]. Fifteen mg of essential oil were dissolved in 15 mL of 2% DMSO and from this solution 5, 50 and 500 μL were transferred to flasks (three flasks for each concentration), to obtain solutions containing 10, 100 and 1,000 μg/mL, respectively. The solvent was allowed to evaporate overnight under sterilized condition in a laminar flow hood. Twenty mL of a medium of the following composition: KH_2_PO_4_ (680), KNO_3_ (1515), Ca(NO_3_)_2_·4H_2_O (1180), MgSo_4_·7H_2_O, H_3_BO_3_ (2.86), MnCl_2_·4H_2_O (3.62), FeCl_3_·4H_2_O (5.40), ZnSO_4_·5H_2_O (0.22), CuSO_4_·5H_2_O (0.22), Na_2_MoO_4_·2H_2_O (0.12), EDTA (11.20), were added to each flask. Other flasks (three for each test) were supplemented with the medium alone, to serve as a negative control. To each flask ten plants of *L. minor* with 2–3 fronds were transferred. The flasks were kept under about 12 h day light conditions. Plants were observed daily and on the seventh day the numbers of fronds were counted. 

### 3.7. Cytotoxicity

The cytotoxic activity of the essential oil of was tested using the brine shrimp assay as previously reported [34]. About 20 mg of essential oil were dissolved in 2 mL 2% DMSO and from this solution 5, 50 and 500 μL were transferred to vials (three vials/concentration), to obtain final concentrations of 10, 100 and 1000 μg/mL, respectively. The solvent was allowed to evaporate overnight. Five mL of a seawater solution (38 g/L) were added to each vial. After 36 h of hatching and maturation of larvae as nauplii, 10 larvae were transferred to each vial using a Pasteur pipette. The vials were then placed at room temperature (25–27 °C) under illumination. Other vials were supplemented with brine solution and served as a negative control.

### 3.8. Animals

Rabbits of either sex with (1.0–1.4 kg) were used. The animals were kept for 14 days before starting the experiments at the Animal House of the Department of Pharmacy, University of Malakand (Lower Dir, Pakistan), under standard conditions mentioned in the *Animals Bye-Laws 2008 of the University of Malakand (Scientific Procedures Issue-1)* and fed on standard diet and tap water. The animals were kept in fasting condition 24 h prior to the start of the experiments with free excess to water. 

### 3.9. Antispasmodic Activity

Experiments on rabbit’s jejunum preparations were carried as previously reported [35]. Slaughtered animals were dissected to open the abdomen and jejunum portion(s) were extracted and kept in freshly prepared Tyrode’s solution, aerated with Carbogen to keep them alive and ready for use. Quiescent sub-maximal doses of acetylcholine (0.3 μM) were used when needed for keeping the tissue viable and alive. A portion of about 1.5 cm length tissue was mounted in a 10 mL tissue bath containing Tyrode’s solution and stabilized for 25–30 min. All the processes were carried out at 37 ±1 °C with constant aeration and kept under 1 *g* pressure. On attaining a reproducible response, test samples at the doses of 0.01, 0.03, 0.1, 0.3, 1.0, 3.0, 5.0, and 10.0 mg/mL essential oil were applied to the bath solution. The experiments were repeated thrice. For the determination of the possible mode of action, the tissue was pretreated with a high concentration KCl (80 mM) to cause depolarization and keep the tissue in sustained contraction. The essential oil was then applied in cumulative manner to obtain a dose dependent curve and relaxation. Intestinal responses data were recorded using a Force Transducer (MLT 0210/A, Pan Lab. S.I., Letica Scientific Instruments*,* Barcelona, Spain) attached with a Power lab (4/25 T, AD Instruments, Bella Vista, New South Wales, Australia). Data were recorded at a range of 20 mv. 

### 3.10. Statistics and Interpretation

Chart 5 (AD Instruments) was used to interpret the graph tracings. Student *t* test was used at 95% confidence interval. *p* values less or equal to 0.05 were considered as statistically significant.

## 4. Conclusions 

The data obtained contribute to a phytochemical knowledge of the essential oil of an important flavor species, widely used in traditional medical practices. The chemical pattern of the essential oil agrees with the composition of other *Skimmia* essential oils. The oil showed antibacterial activity against some of the tested bacterial strains and such activity could be, at least in part, explained by the high percentage of linalyl acetate. On the other hand, the essential oil showed a good antifungal activity against some strains. Of interest seems the activity against *Candida albicans*, considering the pathogenicity of this microorganism. Data about the phytotoxicity of the essential oil agree with the available literature, that ascribes to the volatile fractions antigerminative and allelopathic properties. The muscle relaxation caused by *S. laureola* essential oil suggests that the possible mode of action is either by blocking the release of stored calcium from the sarcoplamic reticulum or by blocking the calcium channels. The data obtained can be useful in explaining the traditional uses of the plant.

## References

[1] Hamayun M. (2007). Traditional uses of some medicinal plants of Swat Valley, Pakistan. Indian J. Tradit. Knowl..

[2] Bhattarai S., Chaudhary R.P., Taylor R.S.L. (2006). Ethnomedicinal plants used by the people of Manang District, central Nepal. J. Ethnobiol. Ethnomed..

[3] Qureshi R.A., Ghufran M.A., Gilani S., Yousaf Z., Abbas G., Batool A. (2009). Indigenous medicinal plants used by local women in Southern Himalayan regions of Pakistan. Pak. J. Bot..

[4] Shah W.A., Qurishi M.A., Thappa R.K., Dhar K.L. (2003). Seasonal variation in the essential oil composition on *Skimmia laureola*. Indian Perfurmer.

[5] Shah W.A., Dar M.Y., Kuratull-Ai, Rather M.A., Qurishi M.A. (2013). Comparison of terpene composition of *Skimmia laureola* using hydrodistillation and HS-SPME techniques. J. Essent. Oil Bear. Plants.

[6] Shah W.A., Dar M.Y., Zagar M.I., Agnihotri V.K., Qurishi M.A., Singh B. (2013). Chemical composition and antimicrobial activity of the leaf essential oil of *Skimmia laureola* growing wild in Jammu and Kashmir, India. Nat. Prod. Res..

[7] Jangwan J.S., Kumar N., Singh R. (2010). Analysis of composition and antibacterial activity of essential oil of *Skimmia laureola* from Garhwal, Himalaya. Internat. J. Chem. Sci..

[8] Mathela C.S., Melkani A.B., Pant A.K. (1992). Reinvestigation of *Skimmia laureola* essential oil. Indian Perfumer.

[9] Sultana N., Sultana R. (2009). A new lanostane triterpene from *Skimmia laureola*. Z. Naturforsch. B.

[10] Atta-ur-Rahman, Khalid A., Sultana N., Ghayur M.N., Mesaik M.A., Khan M.R, Gilani A.H., Choudary M.I. (2006). New cholinesterase inhibiting and calcium blocking alkaloids. J. Enzym. Inhib. Med. Chem..

[11] Sultana N., Afaza N., Atta-ut-Rahman (2004). Coumarin and quinoline alkaloids from *Skimmia laureola*. Sci. Internat..

[12] Mehmood F., Manzoor F., Khan Z.D., Ali M.I., Khan I., Rahim S.M.A. (2012). Evalutaion of toxicity and repellency of essential oil of family Rutaceae against black ants (*Lasius niger*) in Pakistan. Asian J. Chem..

[13] Muhammad N., Barkatullah, Ibrar M., Khan H., Saeed M., Kahn A.Z., Kaleem W.A. (2013). *In vivo* screening of essential oils of *Skimmia laureola* leaves for antinociceptive and antipyretic activity. Asian Pac. J. Trop. Biomed..

[14] Sultana N., Atta-ur-Rahman, Khan T.H. (2005). Tyrosinase inhibitor fatty ester and a quinoline alkaloid from *Skimmia laureola*. Z. Naturforsch. B.

[15] Barkatullah, Ibrar M., Muhammad N., Rauf A. (2013). Antipyretic and antinociceptive profile of leaves of *Skimmia laureola*. Middle-East J. Sci. Res..

[16] Ahmad K.F., Sultana N. (2003). Studies on bioassay directed antifungal activity of medicinal plants *Calotropis procera*, *Skimmia laureola*, *Peltophorum pterocarpum* and two pure natural compounds ulopterol and 4-methoxy-1-methyl-3-(2'S-hydroxy-3'-ene buthyl)-2-quinolone. J. Chem. Soc. Pak..

[17] Riazzudin S., Malik M.M., Nasim A. (1987). Mutagenicity testing of some medicinal herbs. Environ. Mol. Mutagen..

[18] Gondwal M., Prakash O., Vivekanand, Pant A.K., Padalia R.C., Mathela C.S. (2012). Essential oil composition and antioxidant of leaves and flowers of *Skimmia anquetilia* N.P. Taylor & Airy Shaw. J. Essent. Oil Res..

[19] Trombetta D., Castelli F., Sarpietro M.G., Venuti V., Cristiani M., Daniele M., Sajia A., Mazzanti G., Bisignano G. (2005). Mechanism of antibacterial action of three monoterpenes. Antimicrob. Agents Chemother..

[20] Cleff M.B., Meinerz A.R., Xavier M., Schuch L.F., Meireles M.C.A., Rodrigues M.R.A., Mello J.R.B. (2010). *In vitro* activity of *Origanum vulgare* essential oil against *Candida* species. Braz. J. Microbiol..

[21] Pattnaik S., Subramanyam V.R., Bapaji M., Kole C.R. (1997). Antibacterial and antifungal activity of aromatic constituents of essential oils. Microbios.

[22] Hristova Y., Gochev V., Wanner J., Jirovetz L., Schmidt E., Girova T., Kuzmanov A. (2013). Chemical composition and antifungal activity of essential oil of *Salvia sclarea* L. from Bulgaria against clinical isolates of *Candida* species. J. Biosci. Biothechnol..

[23] Tworkoski T. (2002). Herbicide effects of essential oils. Weed Sci..

[24] De Martino L., Mancini E., Marandino A., Rolim de Almeida L.F., De Feo V. (2012). Chemistry and antigerminative activity of essential oils and monoterpenoids from Mediterranean Plants. Curr. Bioactive Comp..

[25] De Martino L., Mancini E., Rolim de Almeida L.F., De Feo V. (2012). The antigerminative activity of twenty-seven monoterpenes. Molecules.

[26] Moshafi M.H., Sharififar F., Dehghan G.R., Ameri A. (2009). Bioassay screening of essential oil and various extracts of fruits of *Heracleum persicum* Desf. and rhizomes of *Zingiber officinale* Rosc. using brine shrimp cytotoxicity assay. Iranian J. Pharm. Res..

[27] Krishnaraju A.V., Rao-Tayi V.N., Sundararaju D., Vanisree M., Tsay H.S., Subbaraju G.V. (2005). Assessment of bioactivity of Indian medical plants using brine shrimp (*Artemia salina*) lethality assay. Int. J. Appl. Sci. Eng..

[28] (2004). European Pharmacopoeia.

[29] Jennings W., Shibamoto T. (1980). Qualitative Analysis of Flavour and Fragrance Volatiles by Glass Capillary Gas Chromatography.

[30] Davies N.W. (1990). Gas chromatographic retention indices of monoterpenes and sesquiterpenes on methyl silicone and Carbowax 20M phases. J. Chromatogr. A.

[31] Adams P. (2007). Identification of Essential Oil Components by Gas Chromatography/Mass Spectrometry.

[32] Goodner K.L. (2008). Practical retention index models of OV-101, DB-1, DB-5, and DB-Wax for flavor and fragrance compounds. LWT-Food Sci. Technol..

[33] (1998). Wiley Registry of Mass Spectral Data, with NIST Spectral Data CD Rom.

[34] Barkatullah, Ibrar M., Muhammad N. (2011). Evaluation of *Zanthoxylum armatum* DC. for *in vitro* and *in vivo* pharmacological screening. Afr. J. Pharm. Pharmacol..

[35] Gilani A.H., Khan A., Jabeen Q., Subhan F., Ghafar R. (2005). Antispasmodic and blood pressure lowering effects of *Valeriana wallichii* are mediated through K^+^ channel activation. J. Ethnopharmacol..

